# Case Report: Serum IgG4-negative multifocal IgG4-related hepatic inflammatory pseudotumor: dissociation between circulating IgG4 and local immune activation

**DOI:** 10.3389/fimmu.2026.1816493

**Published:** 2026-04-15

**Authors:** Wei Wang, Dawei Zhao, Ruichen Ren, Zhongkai Zhou, Jing Chang, Chen Shao, Songtao Liu

**Affiliations:** 1 Department of Radiology, Beijing YouAn Hospital, Capital Medical University, Beijing, China; 2 Department of Pathology, Beijing YouAn Hospital, Capital Medical University, Beijing, China; 3 Department of Medical Oncology, Beijing YouAn Hospital, Capital Medical University, Beijing, China

**Keywords:** IgG4-related disease (IgG4-RD), IgG4-related hepatic inflammatory pseudotumor (IgG4-HIPT), seronegative IgG4-RD, multifocal hepatic lesions, magnetic resonance imaging, differential diagnosis

## Abstract

Immunoglobulin G4-related disease (IgG4-RD) is a systemic immune-mediated fibroinflammatory disorder characterized by aberrant immune regulation and progressive stromal remodeling. Hepatic involvement in the form of IgG4-related hepatic inflammatory pseudotumor (IgG4-HIPT) is uncommon and may radiologically resemble malignant or infectious liver disease. Although elevated serum IgG4 levels are often considered supportive for diagnosis, normal serum IgG4 concentrations do not exclude the disease and may obscure timely recognition. We describe a 60-year-old man presenting with multifocal hepatic lesions, mild inflammatory symptoms, and normal serum IgG4 levels. Imaging revealed arterial phase enhancement, peripheral rim enhancement, diffusion restriction, and increased metabolic activity on ^18F-FDG PET/CT, raising strong suspicion for metastatic disease or hepatic abscess. Histopathological analysis demonstrated dense IgG4-positive lymphoplasmacytic infiltration, storiform fibrosis, and obliterative phlebitis, establishing the diagnosis of IgG4-HIPT. Marked radiologic regression was observed following corticosteroid therapy. This case illustrates a serum IgG4-negative phenotype of IgG4-HIPT and highlights the potential dissociation between circulating immunoglobulin levels and localized immune-driven fibroinflammatory activity. These findings support the concept that IgG4-RD represents an immune-mediated stromal remodeling disorder rather than a purely antibody-dependent condition. Early tissue confirmation is essential to guide immunomodulatory therapy and prevent unnecessary oncologic intervention. Recognition of this entity broadens the understanding of immune-mediated liver disease and underscores the importance of mechanism-based treatment approaches.

## 1 Introduction

Immunoglobulin G4-related disease (IgG4-RD) is a systemic, immune-mediated fibroinflammatory disorder typically characterized by mass-forming lesions. Histologically, it is defined by dense lymphoplasmacytic infiltration enriched with IgG4-positive plasma cells, storiform fibrosis, and obliterative phlebitis (1, 2). Recent studies suggest that the pathogenesis of IgG4-RD involves a process of inflammatory-fibrotic remodeling driven by immune dysregulation, affecting multiple organ systems through organ-specific susceptibility patterns (3).

The hepatobiliary system is a primary site of involvement for IgG4-RD, exhibiting significant clinico-pathological heterogeneity that encompasses IgG4-related sclerosing cholangitis (IgG4-RSC) and mass-forming hepatic inflammatory pseudotumor (HIPT) (4). IgG4-related hepatic inflammatory pseudotumor (IgG4-HIPT) represents a rare focal fibroinflammatory manifestation, considered to be pathologically linked to IgG4-RSC. As the bile duct is a frequent immunological target in IgG4-RD, periductal inflammation and fibrotic remodeling may serve as the mechanistic foundation for hepatic mass formation (5, 6).

Although elevated serum IgG4 is often regarded as a diagnostic clue, its sensitivity is limited; approximately 20–40% of histologically confirmed IgG4-RD cases present with normal serum IgG4 levels (7–9). In recent years, the seronegative phenotype has gained recognition, particularly in localized or single-organ involvement (10–13). However, cases of seronegative IgG4-HIPT presenting with multiple hepatic masses remain sporadic in the literature, representing an exceptionally rare clinical phenotype. Given that its radiological features can closely mimic intrahepatic cholangiocarcinoma, metastatic tumors, or hepatic abscesses, clinical differential diagnosis is highly challenging, making histological evidence the cornerstone of a definitive diagnosis (6, 14–16).

Herein, we report a histologically confirmed case of seronegative, multifocal IgG4-HIPT. While the imaging findings highly simulated malignant or infectious lesions, the patient showed a rapid therapeutic response to corticosteroids. This case provides a clinical paradigm for understanding the immunopathological mechanisms underlying the focal inflammatory-fibrotic processes of IgG4-RD in the liver.

## 2 Case presentation

A 60-year-old male presented with a 19-day history of intermittent fever accompanied by right upper quadrant (RUQ) abdominal pain. The onset was marked by rigors, with a peak body temperature reaching 38.5 °C. Initial laboratory investigations revealed leukocytosis (14.24×10^9^/L) and an elevated C-reactive protein (CRP) level (26.9 mg/L). Following the administration of empirical antimicrobial therapy with cefazolin, the patient’s temperature normalized; however, the RUQ discomfort persisted. Specifically, the patient had no prior history of autoimmune disease, hepatobiliary disorders, or malignancy, and denied any history of chronic alcohol consumption or previous abdominal surgery.

**Figure 1:**
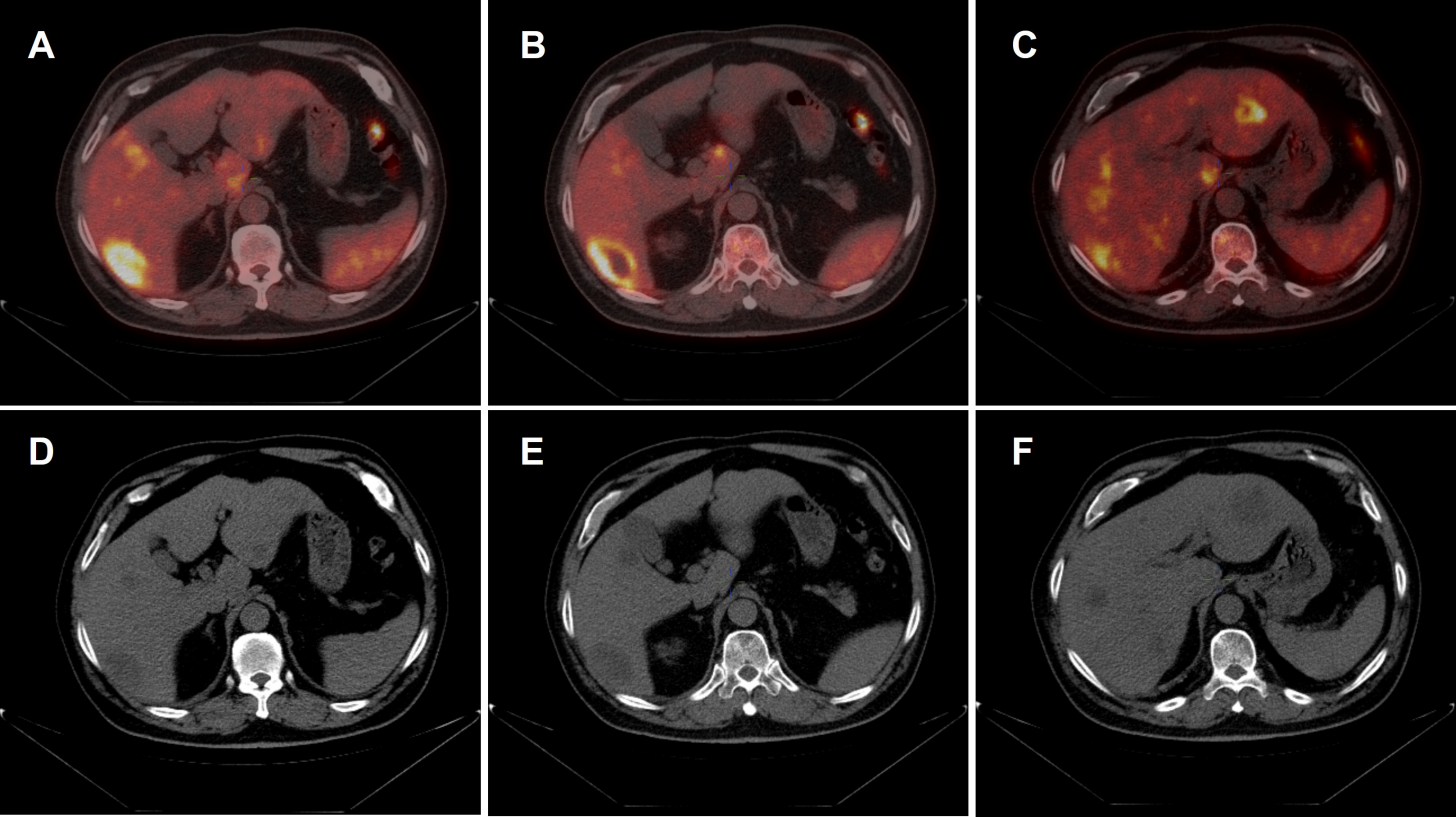
^18F-FDG PET/CT findings. **(A–C)** Fused PET/CT images demonstrate multiple intrahepatic hypermetabolic nodules with ill-defined margins. The largest lesion measures approximately 4.7 × 2.7 cm, with a maximum standardized uptake value (SUVmax) of 9.9. Central photopenic areas are observed in larger lesions, suggestive of internal necrosis. **(D–F)** Corresponding non-contrast CT images show multiple low-density nodules.

**Figure 2:**
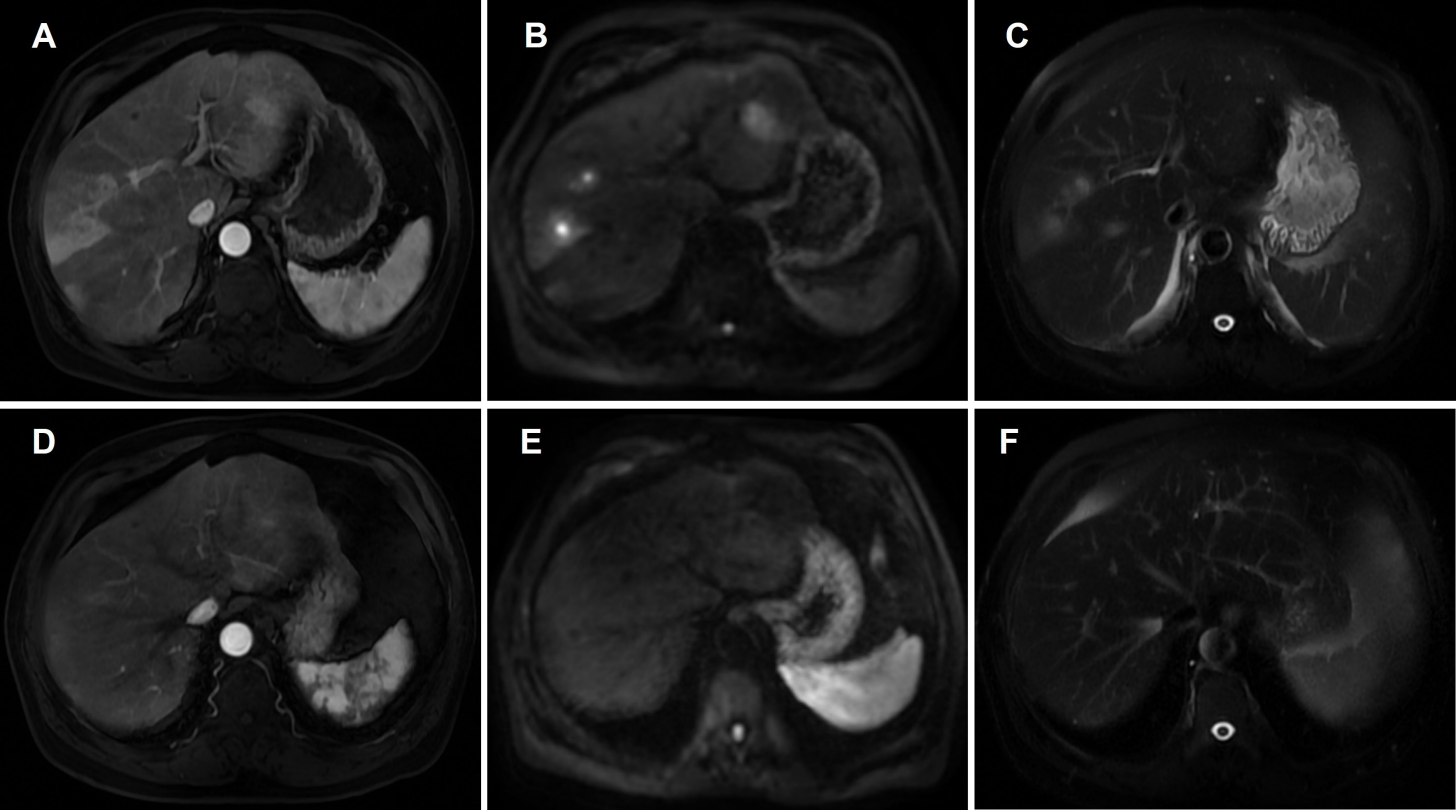
MRI findings before and after corticosteroid therapy. **(A, D)** Contrast-enhanced T1-weighted images. Pre-treatment **(A)** shows multifocal nodular enhancement with peripheral rim-like and patchy arterial enhancement. Post-treatment **(D)** demonstrates near-complete radiological resolution without significant residual enhancement. **(B, E)** Diffusion-weighted imaging (DWI). Pre-treatment **(B)** reveals marked diffusion restriction in multiple hepatic nodules. Post-treatment **(E)** shows marked lesion regression with no apparent diffusion restriction. **(C, F)** T2-weighted images. Pre-treatment **(C)** demonstrates nodules with central high and peripheral slightly high signal intensity, surrounded by perilesional rim-like hyperintensity. Post-treatment **(F)** shows resolution of signal abnormalities.

**Figure 3:**
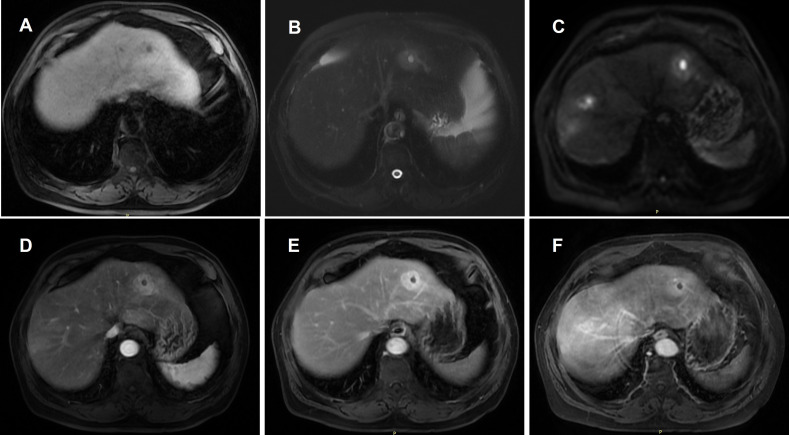
Representative MRI features of multifocal hepatic nodules (left lobe).The lesions exhibit slightly low signal intensity on T1WI **(A)** and slightly high signal intensity on T2WI **(B)**. Central quasi-circular necrotic areas show low T1 and high T2 signal intensity. DWI **(C)** demonstrates diffusion restriction predominantly in the solid components of the lesions. Contrast-enhanced images show mild arterial enhancement **(D)** followed by progressive enhancement in the portal phase **(E)**. The delayed phase **(F)** demonstrates persistent enhancement that remains slightly hyperintense relative to the surrounding liver parenchyma. No enhancement is observed within the necrotic areas across all phases.

Abdominal CT revealed multiple intrahepatic low-density lesions. Upon admission for further evaluation, inflammatory markers remained mildly elevated (CRP 15.44 mg/L), while liver function tests were largely within normal limits (ALT 22 U/L, AST 14 U/L, TBil 9.6 μmol/L). Tumor markers (AFP, CA19-9) were within reference ranges, and screenings for hepatitis B and C viruses were negative. Immunological markers showed an antinuclear antibody (ANA) titer of 1:100. Serum immunoglobulin testing revealed a total IgG of 11.6 g/L and an IgG4 level of 0.16 g/L (reference range: 0.03–2.01 g/L). Contrast-enhanced MRI demonstrated multiple nodular intrahepatic lesions with slightly high signal intensity on T2-weighted imaging (T2WI) and significant diffusion restriction on diffusion-weighted imaging (DWI). Arterial phase imaging showed peripheral rim-like and patchy enhancement, which persisted into the delayed phase with a degree of enhancement slightly lower than the initial phase but higher than the surrounding hepatic parenchyma. Centrally located, quasi-circular non-enhancing necrotic areas were observed within some lesions (**Figures 1A–C**) (**Figure 2**). ^18^F-FDG PET/CT revealed multiple hypermetabolic hepatic nodules with a maximum standardized uptake value (SUVmax) of 9.9, some associated with central necrosis (**Figure 3**). The radiological findings were highly suggestive of hepatic abscesses or metastatic malignancy. Contrast-enhanced MRI and PET/CT showed no evidence of pancreatic enlargement, pancreatic duct irregularity, or bile duct wall thickening, indicating no imaging evidence of pancreatic or biliary involvement. Despite a one-week course of broad-spectrum antibiotic therapy with piperacillin-tazobactam, follow-up imaging showed no significant regression of the lesions.

**Figure 4:**
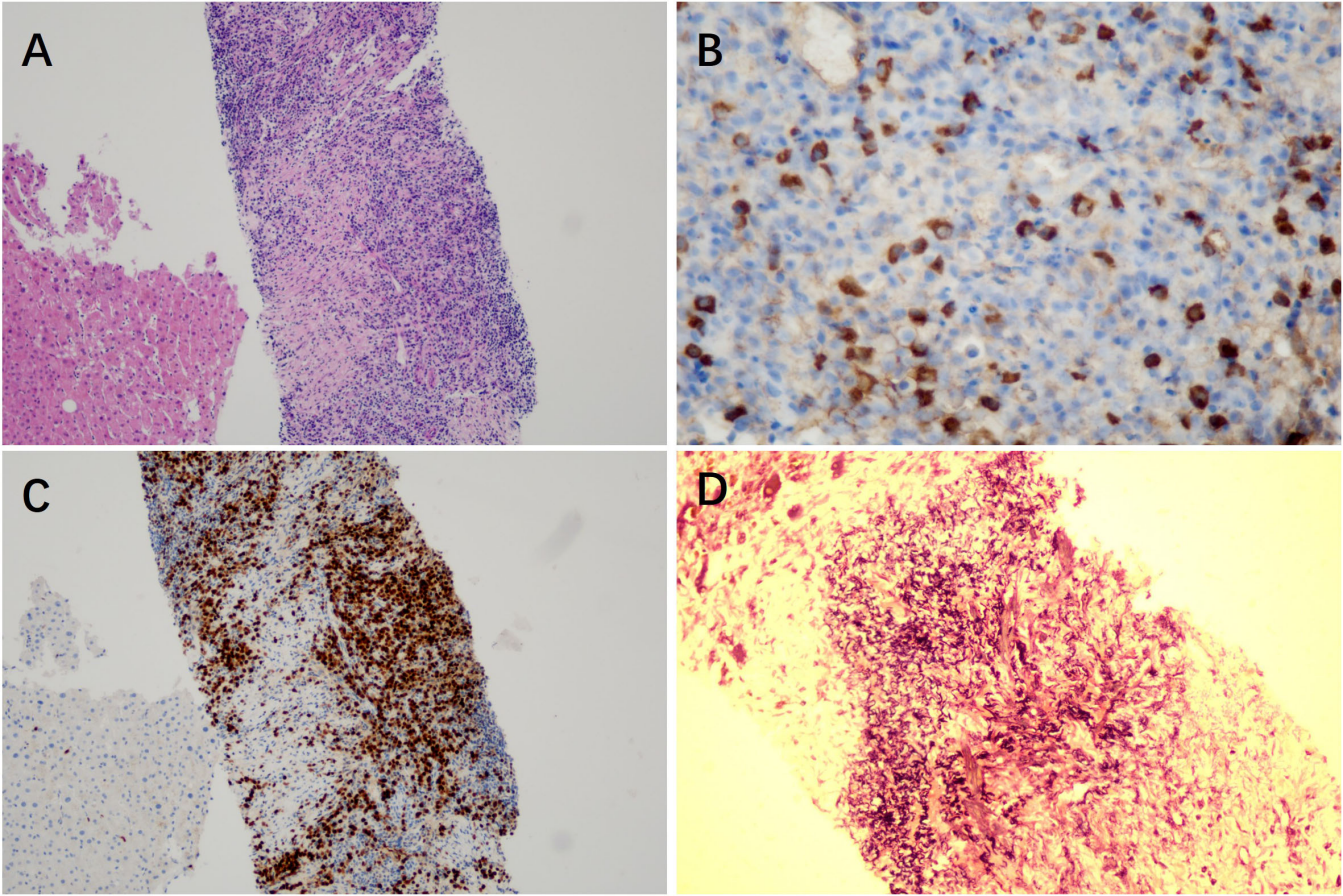
Histopathological findings. **(A)** Liver biopsy showing an inflammatory pseudotumor-like lesion characterized by fibroblast and myofibroblast proliferation with dense lymphoplasmacytic infiltration (hematoxylin and eosin staining, original magnification ×100). **(B)** Immunohistochemical staining demonstrates abundant IgG4-positive plasma cells (IgG4 staining, original magnification ×400). **(C)** Immunohistochemical staining reveals numerous MUM1-positive plasma cells (MUM1 staining, original magnification ×100). **(D)** Elastic fiber staining highlighting obliterative phlebitis, with marked proliferation and disorganized arrangement of elastic fibers in the region of the original portal vein, indicating occlusion of the venous lumen by fibrotic tissue (elastic stain, original magnification ×200).

Given the persistence of the lesions and the radiological ambiguity, an ultrasound-guided percutaneous core needle biopsy of the liver was performed. Histopathological examination revealed dense lymphoplasmacytic infiltration and storiform fibrosis (**Figure 4A**). Elastic fiber staining further demonstrated obliterative phlebitis (**Figure 4D**). Immunohistochemical (IHC) staining demonstrated a marked increase in IgG4-positive plasma cells (>50 per high-power field, HPF), with an elevated IgG4/IgG ratio exceeding 40%. No malignant cells were identified (**Figures 4B, C**). Based on the integration of histological and IHC findings, a definitive diagnosis of IgG4-HIPT was established, despite the normal serum IgG4 levels.

The patient was initiated on oral methylprednisolone at a dosage of 24 mg/day. A follow-up abdominal CT performed two weeks later revealed a significant reduction in the size of the intrahepatic lesions. This indicated a profound therapeutic response to corticosteroid treatment. The dosage was subsequently tapered to 16 mg/day, with concomitant administration of calcium carbonate and omeprazole to prevent corticosteroid-related adverse effects. Upper abdominal MRI performed 50 days later demonstrated further significant remission of the lesions (**Figures 1D–F**), and the methylprednisolone dose was adjusted to 8 mg/day. The patient completed a total of three months of corticosteroid therapy and is currently under active follow-up, with no evidence of recurrence to date.

## 3 Discussion

IgG4-RD is a systemic, immune-mediated fibroinflammatory disorder capable of involving multiple organ systems and manifesting distinct clinical phenotypes characterized by either inflammatory proliferation or fibrotic remodeling (7, 17). Although elevated serum IgG4 levels and mass-forming lesions are traditionally regarded as supportive features, the sensitivity of serum IgG4 is limited; crucially, a normal serum IgG4 level does not preclude a diagnosis of IgG4-RD (7, 8). In hepatobiliary involvement of IgG4-RD, the pancreas and biliary tract are most commonly affected, whereas isolated hepatic involvement has been reported but remains uncommon (6). In the present case, no pancreatic or biliary involvement was identified, further highlighting the diagnostic challenge of IgG4-HIPT. Previous studies have underscored that hepatic inflammatory pseudotumors (IPTs) themselves exhibit substantial clinico-pathological heterogeneity. Specifically, the subtype associated with IgG4-RD can present with complex radiological enhancement patterns and diffusion restriction, showing significant overlap with the features of malignant tumors or infectious hepatic diseases. Consequently, these overlapping features frequently pose a diagnostic dilemma in clinical practice (15, 16). Therefore, when radiological findings remain equivocal, histopathological and IHC evaluations become decisive in establishing a definitive diagnosis of IgG4-HIPT.

The present case was further evaluated according to the 2019 American College of Rheumatology/European League Against Rheumatism (ACR/EULAR) classification criteria for IgG4-related disease, which integrate clinical manifestations, serologic findings, imaging features, and histopathological characteristics within a weighted scoring system (18). The total score in this case exceeded the classification threshold of 20 points (**Supplementary Table 1**), further supporting the diagnosis of IgG4-related disease.

### 3.1 The seronegative IgG4-RD phenotype: diagnostic and biological significance

A hallmark of this case is the seronegativity for IgG4 presenting alongside multifocal hepatic lesions—a clinical constellation that is relatively infrequent in existing literature. Evidence suggests that the seronegative IgG4-RD phenotype may be associated with localized organ involvement and less pronounced systemic immunoglobulin elevation. Furthermore, disease activity and the risk of recurrence in these patients may differ significantly from other clinical subtypes of IgG4-RD (7, 12). Notably, circulating IgG4 levels do not always exhibit concordance with the state of immune activation at the tissue level. Consequently, an over-reliance on serum IgG4 titers as a diagnostic filter may result in substantial diagnostic delays (7, 12, 13). This finding underscores the clinical necessity of obtaining early histological evidence, particularly in cases where radiological suspicion is high or when the therapeutic response to initial management remains atypical.

### 3.2 Immunopathogenetic background

The pathogenesis of IgG4-RD is a complex process of immune dysregulation involving diverse immune cell subpopulations, rather than being directly mediated by the pathogenicity of IgG4 antibodies themselves (19, 20). Follicular helper T (Tfh) cells, particularly the Tfh2 subset, promote IgG4 class switching through the secretion of interleukins IL-4 and IL-10. Simultaneously, cytotoxic T cells and macrophages contribute to the maintenance of chronic inflammation and the progression of fibrosis (19–22). Furthermore, the crosstalk between plasma cells and fibroblasts—exemplified by the PDGF/PDGFR signaling axis—and genetic variants in *IKZF1* and *UBR4* underscore IgG4-RD as a primarily immune-driven fibroinflammatory disorder (23, 24). In the present case, despite normal serum IgG4 levels, the marked infiltration of IgG4-positive plasma cells alongside hallmark histological features was evident. This suggests the presence of a robust, localized, immune-mediated inflammatory response within the hepatic microenvironment.

### 3.3 Radiological pitfalls and differential diagnosis

IgG4-HIPT lacks pathognomonic radiological features, frequently exhibiting characteristics that overlap with malignancies or infectious diseases, thereby complicating clinical diagnosis. Unlike hepatocellular carcinoma (HCC), which typically arises in the setting of chronic liver disease and exhibits the classic “arterial phase hyperenhancement followed by portal/delayed phase washout” pattern (25), IgG4-HIPT often displays progressive enhancement without typical washout. Multifocal lesions characterized by rim enhancement, central liquefaction, and diffusion restriction can closely mimic metastatic disease or hepatic abscesses (26, 27). Furthermore, differentiating IgG4-HIPT from intrahepatic cholangiocarcinoma (ICC) is remarkably challenging, as both entities can exhibit delayed enhancement and biliary involvement, particularly in hilar lesions (28). Although certain features, such as the “duct-penetrating sign” and inflammatory encasement, have been proposed as hallmarks of IgG4-RD, their prevalence is inconsistent, and they show significant overlap with malignant pathologies (28–31). Comparative studies further indicate that MRI alone cannot reliably distinguish between IgG4-related and non-IgG4-related hepatic inflammatory pseudotumor (29, 30). This pronounced radiological overlap reflects an underlying pathology driven by immune-mediated inflammatory fibrosis rather than a classic neoplastic neoangiogenic pattern. (**Table 1**).

**Table 1:** Differential diagnosis of hepatic masses.

Disease	Clinical clues	Typical imaging features	Pathologic features	Key distinguishing points
Hepatocellular Carcinoma (HCC) (25)	Chronic liver disease background; elevated AFP (sometimes)	Arterial hyperenhancement with washout in portal/delayed phase	Malignant hepatocytes with trabecular growth pattern	Washout pattern; cirrhosis background
Intrahepatic Cholangiocarcinoma (ICC) (33)	Often asymptomatic or nonspecific symptoms; may present with biliary obstruction	Peripheral rim arterial enhancement with progressive delayed filling; bile duct dilatation	Adenocarcinoma with desmoplastic stroma	Progressive delayed enhancement and capsular retraction
Hepatic Abscess (34)	Fever; elevated inflammatory markers (CRP/WBC); clinical response to antibiotics	Central liquefaction with thick peripheral rim enhancement (double target sign); marked diffusion restriction with low ADC	Central necrosis with dense neutrophilic infiltration; microorganisms may be identified	Rapid clinical improvement with antibiotics; positive microbiological cultures
Metastatic Liver Tumors (35)	Known primary extrahepatic malignancy	Multiple lesions with continuous peripheral rim enhancement and central necrosis (target/doughnut sign)	Histology consistent with the primary carcinoma; immunophenotype supports site of origin	Multiplicity; continuous peripheral rim enhancement; concordance with known primary tumor
IgG4-Related Hepatic Inflammatory Pseudotumor (IgG4-HIPT) (29)	Mild inflammatory symptoms; serum IgG4 may be elevated or normal	Peripheral arterial enhancement with progressive delayed filling; possible duct-penetrating sign; well-defined margins; diffusion restriction may be present	Dense IgG4-positive lymphoplasmacytic infiltration; storiform fibrosis; obliterative phlebitis	Tissue IgG4 positivity; steroid responsiveness; duct encasement rather than destruction
Non-IgG4 Hepatic Inflammatory Pseudotumor (36)	Variable inflammatory symptoms; may be associated with infection	Ill-defined margins; heterogeneous or peripheral enhancement; central non-enhancement; less frequent delayed progressive enhancement	Inflammatory infiltrate without significant IgG4-positive plasma cells; absence of storiform fibrosis and obliterative phlebitis	Low IgG4/IgG ratio (<40%); absence of IgG4-RD histologic features; less frequent duct-penetrating sign

IgG4-RD exhibits a marked predilection for the hepatobiliary system, with the bile duct representing one of the most frequently involved anatomical structures. Research suggests that IgG4-HIPT may represent a focal manifestation within the clinical spectrum of IgG4-related sclerosing cholangitis (IgG4-RSC), typically characterized by dense IgG4+ plasma cell infiltration and immune-mediated biliary injury (6, 21). The periductal fibroplasia and lymphoplasmacytic infiltration observed in the present case are highly consistent with this pattern, further suggesting a mechanistic link between IgG4-HIPT and biliary inflammatory processes. This underscores the necessity for rigorous differential diagnosis from intrahepatic cholangiocarcinoma (ICC) in clinical practice. Additionally, a subset of hepatic inflammatory pseudotumor (IPT) cases can be accompanied by bacterial infection, particularly in those exhibiting a neutrophil-predominant inflammatory milieu (1). While this patient’s fever resolved following antimicrobial therapy, the lack of significant lesion regression suggests that infection and immune-mediated inflammation may coexist, or that secondary infection may superimpose upon pre-existing inflammatory foci. Such complexity highlights the critical importance of serial radiological assessment and early tissue biopsy for intrahepatic lesions when the etiology remains uncertain.

### 3.4 The pivotal role of biopsy and therapeutic considerations

Given that serum IgG4 is not a definitive diagnostic indicator and radiological features exhibit significant overlap, histopathology remains the gold standard for establishing a diagnosis of IgG4-RD. Even in the presence of elevated serum IgG4 levels, histological examination is essential to exclude mimicking conditions. This necessity arises from the limited specificity of serum IgG4, as several non-IgG4-related inflammatory disorders may also manifest with IgG4-positive plasma cell infiltration. The hallmark histological features of IgG4-HIPT include dense lymphoplasmacytic infiltration rich in IgG4-positive plasma cells, storiform fibrosis, and obliterative phlebitis, which together constitute the classic histopathological triad of IgG4-related disease (6).

Glucocorticoids remain the mainstay of first-line therapy for IgG4-RD, typically inducing rapid resolution of clinical symptoms and radiological manifestations, as evidenced by the significant therapeutic response observed in the current case (22). However, corticosteroid administration warrants clinical vigilance regarding potential infectious risks, particularly given reported instances where IgG4-HIPT progressed to secondary abscess formation following steroid therapy (11). In cases of relapse or a refractory response to corticosteroids, combination therapy with conventional immunosuppressive agents or B-cell depletion therapy (e.g., rituximab) should be considered (22, 32). Given that the natural history of the seronegative IgG4-HIPT phenotype remains poorly defined, prolonged radiological and clinical surveillance is strongly recommended.

## 4 Conclusion

This case characterizes a manifestation of multifocal IgG4-related hepatic inflammatory pseudotumor (IgG4-HIPT) in the presence of normal serum IgG4 levels. Our findings further broaden the recognized clinical spectrum of this disease and underscore several critical takeaways: (1) Serum IgG4 is not a definitive diagnostic biomarker, as its normality does not preclude a diagnosis of IgG4-RD; (2) Radiological findings alone are insufficient for reliably differentiating IgG4-HIPT from malignant neoplasms or infectious pathologies; (3) The early acquisition of histopathological evidence is paramount for guiding appropriate immunosuppressive therapy and averting unnecessary surgical or medical interventions.

## Data Availability

The original contributions presented in the study are included in the article/**Supplementary Material**. Further inquiries can be directed to the corresponding author.
