# Supplementary material for: Case Report: Serum IgG4-negative multifocal IgG4-related hepatic inflammatory pseudotumor: dissociation between circulating IgG4 and local immune activation
Source: Front Immunol. 2026 Apr 15;17:1816493. doi: 10.3389/fimmu.2026.1816493 (PMC13124980; doi:10.3389/fimmu.2026.1816493)
Supplement: Supplementary file 1 [file Table1.docx]

**Supplementary Table S1**

**Application of the 2019 ACR/EULAR classification criteria for IgG4-related disease in the present case**

| **Domain** | **Criterion** | **Finding in the present case** | **Score** |
| --- | --- | --- | --- |
| Entry criterion | Involvement of a typical organ with features suggestive of IgG4-related disease | Hepatic inflammatory pseudotumor with characteristic histopathological features | Eligible |
| Histopathology | Dense lymphoplasmacytic infiltrate, storiform fibrosis, and obliterative phlebitis | All three features present on liver biopsy | 13 |
| Immunostaining | IgG4-positive plasma cells >50 per high-power field and IgG4/IgG ratio >40% | >50 IgG4+ plasma cells/HPF; IgG4/IgG ratio >40% | 14 |
| Serum IgG4 concentration | Serum IgG4 elevation | Normal serum IgG4 level | 0 |
| Imaging features | Mass-like lesion consistent with organ involvement | Multiple hepatic nodular lesions on MRI and PET/CT | 4 |
| **Total score** |  |  | **31** |
